# Insect immunity: oral exposure to a bacterial pathogen elicits free radical response and protects from a recurring infection

**DOI:** 10.1186/1742-9994-11-23

**Published:** 2014-03-07

**Authors:** Lauri Mikonranta, Johanna Mappes, Minna Kaukoniitty, Dalial Freitak

**Affiliations:** 1 Centre of Excellence in Biological Interactions, Department of Biological and Environmental Science, University of Jyväskylä, P.O. Box 35, Jyväskylä FI-40014, Finland; 2 Centre of Excellence in Biological Interactions, Department of Biosciences, University of Helsinki, P.O. Box 65, Helsinki FI-00014, Finland

**Keywords:** Bacterial resistance, Gram-negative, Immune priming, Immunological loitering, Insect immunity, Reactive oxygen species, *Parasemia plantaginis*, *Serratia marcescens*

## Abstract

**Background:**

Previous exposure to a pathogen can help organisms cope with recurring infection. This is widely recognised in vertebrates, but increasing occasions are also being reported in invertebrates where this phenomenon is referred to as immune priming. However, the mechanisms that allow acquired pathogen resistance in insects remain largely unknown.

**Results:**

We studied the priming of bacterial resistance in the larvae of the tiger moth, *Parasemia plantaginis* using two gram-negative bacteria, a pathogenic *Serratia marcescens* and a non-pathogenic control, *Escherichia coli.* A sublethal oral dose of *S. marcescens* provided the larvae with effective protection against an otherwise lethal septic infection with the same pathogen five days later. At the same time, we assessed three anti-bacterial defence mechanisms from the larvae that had been primarily exposed to the bacteria via contaminated host plant. Results showed that *S. marcescens* had induced a higher amount of reactive oxygen species (ROS) in the larval haemolymph*,* possibly protecting the host from the recurring infection.

**Conclusions:**

Our study supports the growing evidence of immune priming in insects. It shows that activation of the protective mechanism requires a specific induction, rather than a sheer exposure to any gram-negative bacteria. The findings indicate that systemic pathogen recognition happens via the gut, and suggest that persistent loitering of immune elicitors or anti-microbial molecules are a possible mechanism for the observed prophylaxis. The self-harming effects of ROS molecules are well known, which indicates a potential cost of increased resistance. Together these findings could have important implications on the ecological and epidemiological processes affecting insect and pathogen populations.

## Introduction

Recurring infections are common in the natural environment. Antibody based immunological memory has evolved in jawed vertebrates to cope with the threat of multiple infections. Invertebrates, being relatively short lived, lack antibodies [1]. However, evidence of insects being protected from pathogens they have previously encountered, has accumulated during the past decade e.g. [2-6]. The phenomenon has been coined as immune priming, and advances in insect immunity have shown that the innate and adaptive systems might be functionally closer to each other than previously thought [7,8].

Development, upregulation, and long-term maintenance of the innate immunity come with fitness costs that can be seen in various life-history traits [2,9-12]. A balance between the costs and the benefits of defences must give a selective advantage to individuals that have the optimal level of protection against the pathogens they are likely to encounter [3,13,14]. The protection could be achieved by a mechanism that allows enhanced reactivation of certain immune defences if the host faces a recurring infection, akin to vertebrate immune memory [4]. Alternatively, it might be beneficial to simply stay prepared for a recurring immune insult after the first encounter with immune elicitors or anti-microbial molecules that can remain stably expressed in the haemolymph [1-3]. The first encounter would serve as a cue for a threat of infection and upregulate the appropriate repertoire of defensive molecules [2,3,13]. This kind of ‘immunological loitering’ [3,15] might be considered as just a coincidental side effect of the primary pathogen detection, but we argue that there are reasons to assume that it is an adaptive trait. If non-infective pathogens can be detected before they become infective, the beneficial effect would be similar to density dependent prophylaxis, [16] where higher density of conspecifics indicates a higher risk of parasite encounter. There is ample evidence that many insects can maintain high levels of various immune molecules in their haemolymph for up to 44 days after immune induction [17-24]. Thus, taking the costs into account, it is hard to believe that this kind of prolonged immune reaction could have evolved without fitness benefits [2].

The anti-microbial mechanisms that insects use immediately when infections occur are relatively well known [25]. The detection of invading bacteria by gram-negative binding protein and peptidoglycan recognition protein leads to the activation of Imd and Toll signalling pathways that induce humoral and cellular responses, providing insects with coarse immunological specificity. These pathways can induce the release of bactericidal reactive oxygen species (ROS), different anti-microbial peptides and specialised haemocytes that also control melanisation and phenoloxidase (PO) activity [7,25-28]. At the same time, both PO and ROS related responses are considered to have high costs as they are accompanied with autoimmune effects [29,30]. Although some good explanations, like phagocytosis, controlled by the Toll pathway [7,31,32] have been proposed, the mechanisms behind priming against an infection occurring later in life, or even in subsequent generations, remain largely unknown [4,31,33].

In this paper we report how midgut mediated immune priming occurs in wood tiger moth *Parasemia plantaginis* (Linnaeus 1758) larvae against an environmental opportunistic bacterial pathogen. We primed the larvae, by exposing them orally to a non-infective dose of pathogenic *Serratia marcescens* and to a similarly gram-negative but non-pathogenic control bacterium *Escherichia coli*. We then assessed the consequences of primary oral encounter with the bacteria in two ways: first, indirectly by measuring the level of immunocompetence (PO, lytic, and ROS activity) from the larval haemolymph five days after the initial oral exposure, and then directly by measuring the survival after a severe secondary septic infection. A sublethal oral dose of *S. marcescens* provided the larvae with resistance against an otherwise lethal septic infection but the non-pathogenic control bacterium failed to confer protection. Priming with *S. marcescens* also induced a higher amount of ROS in the larval haemolymph, an antimicrobial defence that persisted until the secondary infection. This finding offers a potential, novel mechanistic explanation for acquired resistance in insects. Additionally, the activation of the protective mechanism seems to require more specific induction than a sheer exposure to any gram-negative bacteria, suggesting systemic pathogen recognition via midgut.

## Results

**Figure 1:**
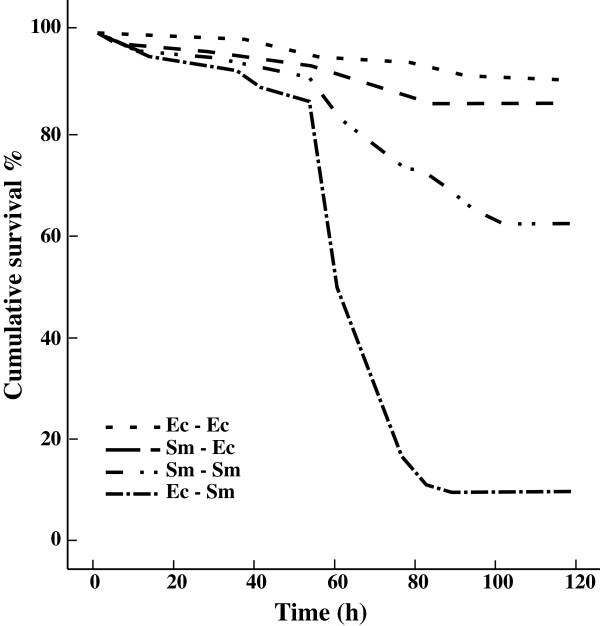
**Larval survival after septic injury.** The four survival curves present different priming-injection groups, where the first term is bacteria used in the priming, and second term bacteria used in septic infection 120 h later. Ec stands for the control bacterium, *E. coli*, and Sm for *S. marcescens*. Treatments from the least to the most virulent combination: Ec-Ec < Sm-Ec < Sm-Sm < Ec-Sm.

Larval survival was significantly affected by the interaction between priming (1^st^ exposure) and injection (2^nd^ exposure) treatments (priming, df = 1 Wald = 1.1, p = 0.290; injection, df = 1, Wald = 64.3, p < 0.001; priming × injection, df = 1, Wald = 15.1, p < 0.001). This indicated that larvae survived the injection differently depending on the previous oral priming. The four priming-injection groups (df = 3) were further compared using pairwise Kaplan-Meier log-rank statistics (Table 1). Larvae injected with the control bacterium showed very low mortality and did not differ from each other regardless of the priming (*Serratia*-*control*: 13.8% mortality and *control-control*: 9.1% mortality). Larvae injected with the pathogenic *S. marcescens* experienced only moderate mortality if they had been previously primed with it (*Serratia-Serratia*: 37.4%), but very high mortality if primed with the control (*control-Serratia*: 90.4%) (Figure 1). There was altogether less than 5% background mortality among the larvae during the priming and no difference between the groups (data not shown).

**Table 1:** Pairwise differences in larval mortality between the priming-injection treatments

**Priming-injection**	***Serratia-control***		***control-Serratia***		***control-control***	
	***χ***^**2**^	**Sig.**	***χ***^**2**^	**Sig.**	***χ***^**2**^	**Sig.**
*Serratia-Serratia*	12.641	**<0.001**	64.92	**<0.001**	19.72	**<0.001**
*Serratia-control*			115.38	**<0.001**	1.07	0.300
*control-Serratia*					130.57	**<0.001**

The larvae were primed orally with either a non-infective dose of pathogenic *Serratia marcescens* or a non-pathogenic control bacterium *Escherichia coli,* and five days later injected with the same or different bacteria.

Statistically significant pairwise differences are bolded.

**Figure 2:**
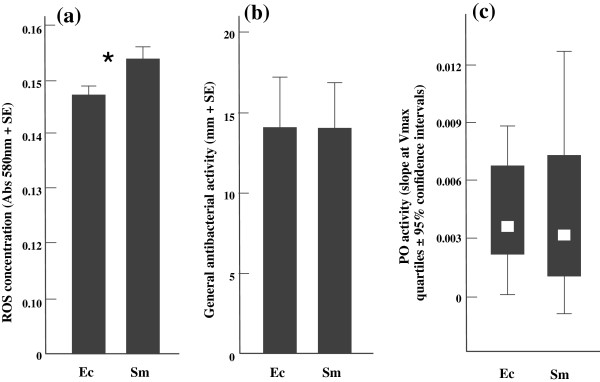
**The immune assays from differently primed larvae 120 h after the initial exposure.** Ec stands for the control bacterium, *E. coli,* and Sm for *S. marcescens*. Asterisk (*) indicates a significant (<0.05) difference between the oral priming treatments. The amount of ROS in the haemolymph **(a)**, the lytic activity **(b)** and the PO activity **(c)**.

Larvae that were primarily exposed to *S. marcescens* had 4.8% higher ROS concentration in their haemolymph compared to priming with the control (df = 21, t = -2.43, p = 0.026; Figure 2a). The lytic activity and PO activity did not differ between the two treatments (Lytic: df = 20, U = 54.50, p = 0.679; PO: df = 21, t = 0.17, p = 0.987; Figure 2b & c).

## Discussion

Here we show that a previous oral exposure to *S. marcescens* protects *P. plantaginis* larvae from an otherwise lethal septic infection with the same pathogen. As a response to the priming with *S. marcescens* the moth larvae also showed elevated levels of reactive oxygen species in their haemolymph five days post-treatment, offering a potential explanation for the protection. The elevated ROS levels were measured prior to the secondary immune challenge, which suggests that the mechanism for the prophylaxis could be due to immunological loitering rather than enhanced capacity to re-upregulate immune defences. This is in agreement with earlier studies, which show that different immune molecules can remain in the hemolymph days or even weeks after the immune challenge [22]. It might be that this simple kind of acquired resistance is more prevalent in short lived insects than generally acknowledged, and serves as a natural "vaccination" if pathogens are first detected in sub-lethal doses. The results do not rule out the possibility that insects could, after down regulation, reactivate a stronger immune reaction against a pathogen they have already encountered, which would be functionally more analogous to immunological memory than a simple persistent immune response. It is, for example, possible that immunological loitering and more "memory-like" functions act in concert to fight recurring infections [1,4]. It could also be that some other molecules, such as antimicrobial peptides Cecropins or Gloverin, that we did not measure are up regulated with ROS and that the persistent protection is not solely due to oxidative defence [28].

*S. marcescens* is very common in the environment, e.g. soil, water and plants, and is often isolated from many insect species across various taxa [34,35]. Thus*,* it is likely that *P. plantaginis* falls within *S. marcescens*’ natural host range and real life encounters via contaminated host plants are possible in the wild. It has been shown that a septic infection with *Serratia* in insects can occur in the wild via, for example, an ovipositor of Hymenopteran parasite [36], a nematode vector [37], or a spontaneous gut rupture [34]. It would be of great benefit for the host to be prepared in advance for such a sudden and intensive immune insult.

We detected higher levels of ROS from the hemolymph of *S. marcescens*-exposed larvae compared to the control group fed with the non-pathogenic *E. coli*. This is a well-known anti-microbial defence mechanism in insects [25], and could have mediated the higher survival when the larvae were infected again with the pathogen. However, the ROS defence alone might not be sufficient to control large doses of *S. marcescens* in septic injury. This is because the bacterium is known to be fairly tolerant against oxidative stress via production of cellular catalases [38]. In addition, ROS are usually thought to control gut microbiota, and might not directly protect against injected pathogens [28,39]. The increased levels of ROS in our study, however, were measured straight from the haemolymph sample. Thus, higher levels of ROS may initially help keep the septic infection under control until other aspects of immunity can diminish it; and/or ROS mediates the regulation of other antimicrobials, such as Diptericin in Drosophila [40].

Interestingly, we did not find correlations between the measured immune traits although several previous studies suggest negative genotypic and phenotypic correlations between different defences [41-43]. For example, encapsulation and lytic activity, which might be targeted against different invaders, have been shown to correlate negatively in a field cricket, *Gryllus bimaculatus*[44]. Two major immunocompetence measures, PO and lytic activity, have been criticized by claiming that these indicators do not predict resistance against a challenge with natural parasites [45,46]. Our findings show ROS being upregulated in the pathogen challenged group, whereas PO and lytic activity show no change. However, the upregulation of different immune pathways are most likely pathogen and host specific. Indeed, it has been shown that *Daphnia magna* with higher induced PO levels are more resistant to their parasite *Pasteuria ramosa*[24]. Our observation of the lack of negative relationships within the immune system traits does not mean that the ROS response would be trade-off free. The major cost of resistance, in this case, could result from the non-specific nature of ROS molecules that are well known to cause self-harm and early senescence [30,40,47]. This could mean that prolonged exposure to the free radicals in the haemolymph requires additional resources to deal with potential tissue damage. Also, pathogen induced persistent immune reactions could have adverse effects on the native gut flora, indirectly contributing to fitness consequences [48].

Given the obvious costs, hosts should avoid unnecessary upregulation of immune responses. Imd-pathway mediated immune defence is often thought to be activated by the presence of peptidoglycan fragments from any gram-negative bacteria [25]. Nehme et al. [27] also proposed this to be the case between Drosophila and *S. marcescens* in septic infection. However, even the highly virulent *S. marcescens* db11-strain did not elicit an immune response via the oral route in that particular system. The defence observed in this paper must have been triggered by a more specific mechanism than a general response to the presence of gram-negative bacteria in the gut because priming with the control bacterium failed to confer the protection. Both bacteria exhibit DAP-type peptidoglycan in their cell wall, which has been shown to activate the Imd-pathway, in contrast to Lys-type found in gram-positives and the Bacillus group [49]. It has been suggested that in Drosophila larvae, haemocytes in the gut can signal pathogen presence to the fat body, via cytokines or by releasing ingested cell wall fragments [50,51]. It is thus possible that *P. plantaginis* haemocytes can recognize potentially harmful *S. marcescens* and ignore benign bacteria*.* Also, bacterial immune elicitors (e.g. peptidoglycan or lipopolysaccharides) are known to bind to a storage and transport protein vitellogenin in fish [52,53]. It is also a very abundant protein in the insect hemolymph [54] and, interestingly, has antioxidative capabilities protecting organisms against free radical stress [55]. In another lepidopteran, *Manduca sexta,* direct inoculation of *E. coli* in the haemocoel has been shown to offer resistance against *Photorhabdus luminescens* via upregulation of pattern recognition proteins [56]. The seemingly contradictory results with our experiment probably stem from the priming method: if introduced orally, *E. coli* most likely does not penetrate the gut epithelium, nor it is beneficial for the host to actively present antigens from a non-pathogenic bacteria to the fat body or haemolymph [50,51]. In our study, *S. marcescens*, but not *E. coli*, offered the protection and elicited the systemic ROS response in the haemocoel when detected in the gut. The different result with septic first exposure thus provides further support for the intestinal recognition of pathogenic and non-pathogenic bacteria and for the immune systems ability to mount a corresponding systemic defence. Another alternative explanation is that our non-pathogenic control bacterium appears in the gut in quantities that do not exceed the detection threshold of the recognition proteins, compared to *S. marcescens* that might still proliferate in the gut even when being avirulent [27]. Regardless of the mechanism, a harmless encounter with a pathogen via gut transfers into a systemic immune reaction that protects *P. plantaginis* larvae when substantial amount of the same pathogen is introduced straight into haemocoel later in life.

## Conclusions

A lepidopteran species, although having a relatively short life span, remains protected against a previously encountered pathogen, possibly because of persistent immune responses involving free radicals. The findings evoke interesting questions on the evolutionary and epidemiological consequences that priming might have in insect populations, through increased resistance, and on the other hand, through increased costs due to oxidative stress. Although the ecological and evolutionary effects of priming are very hard to study at the population level in the wild, modelling suggests that it has evident consequences on both pathogen (disease prevalence) and host (demographic structure) population dynamics, as well as on the stability of host-parasite systems [57,58].

## Material and methods

### Study species

*P. plantaginis,* the wood tiger moth, is a day active moth distributed over the northern hemisphere. It has been extensively studied for its warning coloration [59-61]. Also, a few studies describe moth immunocompetence, and interaction between larvae and *S. marcescens.* For example, larval diet has been shown to have a substantial effect on the level of immune defence [62-64]. Larvae used in this experiment were obtained from a population originating from wild individuals caught in southern Finland and kept for three generations in the laboratory (see methods for rearing in [61]).

*S. marcescens* is a cosmopolite opportunistic pathogen that is commonly found in water and soil. It causes nosocomial infections in humans and has been isolated from various insect species [34,35,65]. The strain used in the experiment was obtained from American Type Culture Collection (ATCC# 13880). Laboratory adapted *E. coli* K-12 was used as the non-pathogenic control strain. The bacteria were maintained in standard LB-medium (10 g tryptone, 5 g yeast extract, 10 g NaCl in 1 L of dH_2_O).

### Priming and injection

416 three-week-old moth larvae were weighed, after which they were randomised to the two primary exposure treatments (*S. marcescens,* N = 207 and *E. coli,* N = 209). *E. coli* was used as a control treatment instead of a completely naïve group because we wanted to see how the sheer presence of gram-negative bacteria in the diet would compare to the actual pathogen. It has been shown previously that in spite of being non-pathogenic, the presence of *E. coli* may induce a general immune reaction in insects e.g. [41, 66]. The larvae were placed individually on Petri dishes and reared at 21°C under a 15 hour light: 9 hour dark cycle. Larval weight did not differ between treatments (df = 414, t = -0.6, p = 0.55). Larvae were first fed with their natural diet, dandelion (*Taraxacum sp*), after which it was supplemented with the priming cultures. The bacterial mass was grown overnight on LB-agar plates in 31°C, scraped off with sterile loops and mixed to liquid LB. To standardise the amount of cells the mass was diluted until 0.50 optical density (OD) at 600 nm was reached. These dilutions were then added to the larval diet by pipetting a 200-μl droplet (approximately 6 × 10^7^ cells) of the priming solution to each dandelion leaf surface. After 48 h, majority of the larvae had consumed all the contaminated food and they were given normal diet again. 120 h after the primary priming exposure larvae were infected by injecting 2 μl (OD 0.16, 90 000?*S. marcescens* and 110 000 *E. coli* cells) of bacteria (the previously encountered pathogen, or the control bacterium) directly into the body cavity. The injection was given behind the fifth proleg with a 10 μl Hamilton syringe. The larvae were kept under constant conditions with *ad libitum* food and survival was recorded every three hours. We took haemolymph samples from 15 random larvae (not included in the survival analysis) per priming treatment before the injection.

### Immune assays

PO and ROS activities were estimated from samples containing 10 μl of larval haemolymph diluted in 30 μl ice-cold potassium phosphate buffer which was then frozen at -80°C. For measurements, the samples were thawed and centrifuged (9000 g) at 4°C for 10 minutes to obtain the clear supernatant. For PO, 25 μl of supernatant was added to 200 μl of 3 mM L-Dopa (Sigma, #333786). Kinetic activity of the enzyme was measured at 30°C, 490 nm for 90 minutes (1 minute intervals) with Victor X4 2030 plate reader (Perkin Elmer, Waltham, MA, US). The slope of the absorbance curve from 10–80 minutes was used in the analyses [41].

Pierce PeroXOquant™ quantitative peroxide assay kit (Thermo Scientific, Waltham, MA, US #23280) was used to estimate the amount of ROS in the haemolymph: 5 μl of the supernatant was mixed with 90 μl of the manufacturers working solution. Eight dilutions (ranging from 1 to 1000 μM) of H_2_O_2_ were used as standards. The mix was left to stabilize at room temperature for 25 min after which absorbance was read with a Bioscreen™ spectrophotometer (Growth Curves Ltd., Helsinki, Finland) at 580 nm.

Lytic activity was assessed straight from the haemolymph samples by pipetting 5 μl of fresh haemolymph into a 2.2 mm diameter wells punctured on *Micrococcus* (ATCC #4698) agar plate, incubated over night in 31°C and then photographed. 7 serial dilutions (0.031 - 2.0 mg/mL) of lysozyme (Sigma, #L7651) were used as standards. Lytic activity was measured from the photo as the diameter of a degradation halo around the well [41].

### Statistical analyses

Larval survival was first analysed using Cox-regression with priming, injection, and their interaction in the model. The four priming-injection groups (*Serratia*-*control, control-control*, *Serratia-Serratia* and *control-Serratia*) were then compared with pairwise Kaplan-Meyer survival analysis. PO activity and ROS were analysed with a t-test. Mann–Whitney U-test was used for lytic activity because of non-normal distribution. All analyses were performed with SPSS statistics 21.0.

## Competing interests

The authors declare that they have no competing interests.

## Authors’ contributions

LM, JM, MK, and DF designed the experiment and carried out the experimental work. LM and DF analyzed the data. LM, JM, and DF wrote the manuscript. All authors read and approved the final manuscript.
